# Microbiome-Metabolome Signature of Acute Kidney Injury

**DOI:** 10.3390/metabo10040142

**Published:** 2020-04-04

**Authors:** Nadezda V. Andrianova, Vasily A. Popkov, Natalia S. Klimenko, Alexander V. Tyakht, Galina V. Baydakova, Olga Y. Frolova, Ljubava D. Zorova, Irina B. Pevzner, Dmitry B. Zorov, Egor Y. Plotnikov

**Affiliations:** 1 Faculty of Bioengineering and Bioinformatics, Lomonosov Moscow State University, Moscow 119992, Russia; 2 A.N. Belozersky Institute of Physico-Chemical Biology, Lomonosov Moscow State University, Moscow 119992, Russia; 3 V.I. Kulakov National Medical Research Center of Obstetrics, Gynecology and Perinatology, Moscow 117997, Russia; 4 Atlas Biomed Group - Knomics LLC, Skolkovo Innovation center, Moscow 143026, Russia; 5 Center for Precision Genome Editing and Genetic Technologies for Biomedicine, Institute of Gene Biology, Russian Academy of Sciences, Moscow 119334, Russia; 6 Research Centre for Medical Genetics, Moscow 115522, Russia; 7 Institute of Mitoengineering MSU» LLC, Leninskiye Gory 1, 119192 Moscow, Russia; 8 Sechenov First Moscow State Medical University, Institute of Molecular Medicine, Moscow 119991, Russia

**Keywords:** acute kidney injury, microbiota, fecal bacteria, 16S rRNA gene sequencing, metabolites, bacterial balances, creatinine, urea

## Abstract

Intestinal microbiota play a considerable role in the host’s organism, broadly affecting its organs and tissues. The kidney can also be the target of the microbiome and its metabolites (especially short-chain fatty acids), which can influence renal tissue, both by direct action and through modulation of the immune response. This impact is crucial, especially during kidney injury, because the modulation of inflammation or reparative processes could affect the severity of the resulting damage or recovery of kidney function. In this study, we compared the composition of rat gut microbiota with its outcome, in experimental acute ischemic kidney injury and named the bacterial taxa that play putatively negative or positive roles in the progression of ischemic kidney injury. We investigated the link between serum creatinine, urea, and a number of metabolites (acylcarnitines and amino acids), and the relative abundance of various bacterial taxa in rat feces. Our analysis revealed an increase in levels of 32 acylcarnitines in serum, after renal ischemia/reperfusion and correlation with creatinine and urea, while levels of three amino acids (tyrosine, tryptophan, and proline) had decreased. We detected associations between bacterial abundance and metabolite levels, using a compositionality-aware approach—*Rothia* and *Staphylococcus* levels were positively associated with creatinine and urea levels, respectively. Our findings indicate that the gut microbial community contains specific members whose presence might ameliorate or, on the contrary, aggravate ischemic kidney injury. These bacterial taxa could present perspective targets for therapeutical interventions in kidney pathologies, including acute kidney injury.

## 1. Introduction

Modern discoveries suggest that the intestinal microbiome (its composition and activity), as well as the gut barrier that restrict the entry of bacteria and their metabolites into the blood and other body tissues, can be compromised in pathologies and have a great influence on many organism functions associated with the immune system [1]. Composition of the microbiome is related with incidence of obesity, diabetes, certain cancers, diseases of the intestine and cardiovascular system, and kidneys pathologies [2,3,4,5,6,7,8]. It has been also shown that a number of diseases, for example, stroke and chronic kidney disease, in turn, lead to alterations in the composition of the microbiota, creating a “vicious circle” [9,10]. Some bacteria can reduce the barrier function of the mucous membrane by producing special lipopolysaccharides and proteases [11]. Intestinal permeability is a very important factor modulating the immune response and affecting other organs, apart from the gastrointestinal tract [12]. The penetration of bacteria through the gut wall leads to the increased presence of microorganisms or their components in the bloodstream, resulting in the activation of the immune system [13]. The concept of “healthy human blood microbiome” has been recently proposed, however, when the levels of bacteria or their derivatives in the bloodstream exceeds a certain threshold, this triggers systemic inflammation and sepsis (SIRS), which negatively affects all organs and tissues [14]. Therefore, it is believed that targeted modulation on microbiota or gut barriers can reduce the immune system activation and inflammatory response [15]. 

Inflammation is a well-known pathogenetic mechanism of renal damage that occurs not only during infection but also as a response to many damaging factors, such as ischemia [16]. While the normal inflammatory reaction is a common component of the tissue stress response, its excessive activation (for example, caused by bacterial intervention) leads to structural and functional disorders in the renal tissue [17]. Particularly, it has been shown that the use of broad-spectrum antibiotics leads to significant alleviation of the severity of acute kidney injury (AKI), indicating the impact of the microbiome on the kidney [18]. Paradoxically, it was found that in germ-free animals, AKI is more pronounced; such animals had a shift in their blood baseline level of cytokines, with a predominance of pro-inflammatory interleukins, as well as a smaller number of regulatory T-cells that control the immune response [19,20]. It was suggested that since the microbiota is necessary for the modulation of immune cells, especially regulatory T-cells, it plays an important role in regulating kidney inflammatory response during AKI [21].

In addition to affecting the immune system, gut microbiota can interact with the kidneys through the production of various compounds, e.g., short-chain fatty acids (SCFAs) [22]. These acids, represented mainly by acetate, propionate, and butyrate, are the major products of the enzymatic breakdown of complex polysaccharides by the bacteria in the large intestine [23]. SCFAs were shown to decrease the inflammatory response, reduce the infiltration of damaged tissue by leukocytes and affect chemotaxis and cytokine production [24,25].

**Figure 1:**
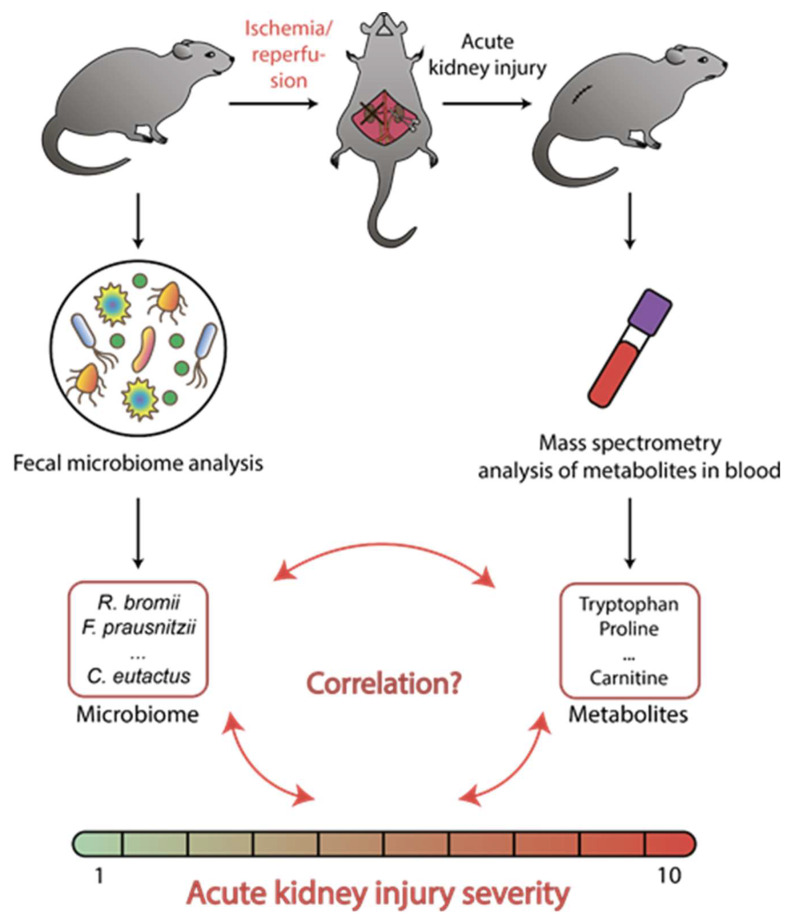
Experimental design. The composition of microbiota was evaluated in fecal samples collected immediately before the modeling of acute kidney injury, as soon as the blood samples were taken after renal ischemia/reperfusion and were analyzed for a number of metabolites (serum creatinine, urea, acylcarnitines, and amino acids).

The aim of this work was to elucidate whether the composition of the gut microbiota could affect the severity of ischemic kidney injury and to identify specific bacterial taxa that likely play negative or positive roles in the progression of kidney damage (Figure 1). We investigated the links between rat intestinal microbiota composition and the AKI caused by ischemia/reperfusion (I/R) of the kidney. The associations of creatinine, urea, and a number of metabolites (amino acids and acylcarnitines) with an abundance of various bacterial taxa were analyzed. 

## 2. Results

### 2.1. AKI and Metabolome

Since ischemic injury is the most common factor leading to AKI [26], we used kidney ischemia/reperfusion (I/R) as a model of AKI. We used a conventional protocol of 40-min unilateral warm ischemia of the left kidney, followed by reperfusion [27]. The analysis was performed on 14 rats subjected to I/R and 6 intact animals. The samples of serum were analyzed for 63 substances. Two substances, namely creatinine and urea, were uremic toxins and serve as common markers of kidney dysfunction, thus, they were used for the estimation of AKI severity. Among other substances, 20 were amino acids and 41 were various acylcarnitines. Datasets of all metabolites levels obtained from the tandem mass spectrometry and biochemical analysis of the serum was provided in Supplementary Table S1.

After I/R, we detected 34 metabolites in blood serum, whose levels significantly changed (Table 1)—concentration of 31 acylcarnitines increased, while the content of 3 amino acids (tyrosine, tryptophan, and proline) dropped. The most significant changes were observed for malonylcarnitine (AC C3DC), which demonstrated a 7-fold increase compared to control, glutarylcarnitine (AC C5DC) (5-fold increase), decadienoylcarnitine (AC C10:2) (4-fold increase), hydroxybutyrylcarnitine (AC C4OH) (4-fold increase), linoleylcarnitine (AC C18:2) (4-fold increase), and methylmalonylcarnitine (AC C4DC) (4-fold increase). Other acylcarnitines showed about a 2-fold increase. The serum levels of the tyrosine, tryptophan, and proline concentration dropped to 60%–70% of their content.

There was a significant correlation between the concentrations of metabolites, which significantly changed after the AKI and the creatinine (reflecting the AKI severity) for 21 metabolites, the absolute Pearson correlation coefficient was higher than 0.7, and for 35 compounds were higher than 0.5 (Table 1). Acylcarnitines had a positive correlation with creatinine, while 3 amino acids had a strong negative correlation with creatinine and urea—tyrosine, tryptophan, and proline had correlation coefficients less than –0.5. The structural formulas of acylcarnitines that, both, showed the most significant change in AKI and had the strongest correlation with the creatinine concentration are shown in the Supplementary Figure S1, along with the possible biochemical pathways involved in their increase.

### 2.2. AKI and Microbiome

We analyzed gut microbiome of each rat by sequencing the V4 region of the 16S rRNA gene for the fecal samples collected before induction of kidney ischemia. Thereafter we compared relative abundance of each bacterial taxon for each rat with the level of serum creatinine. Overall, 1’766’984 reads were obtained during the sequencing (44’492 to 94’776 reads per animal). Generally, the taxonomic composition on the level of genus was dominated by an unclassified genera from the Clostridiales order (20%), *Lactobacillus* (15%) and *Allobaculum* (10%). The detailed composition profiles are available as an interactive online report in the Knomics-Biota system at https://biota.knomics.ru/microbiome-metabolome-sig.

**Figure 2:**
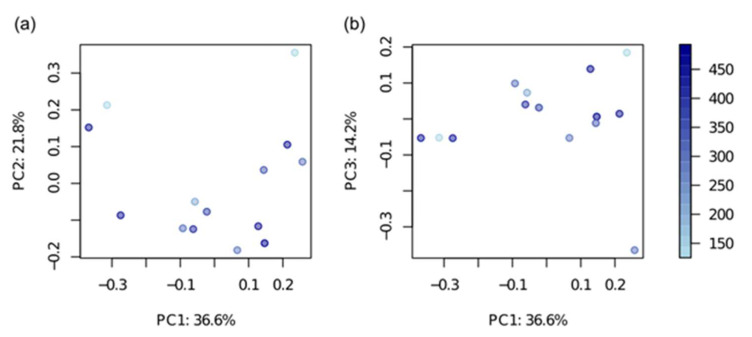
Principal coordinate analysis of bacterial composition on the level of genera. Bray-Curtis diversity metric was used for the distance matrix calculation. The circles are colored according to the creatinine value (from low—light blue, to high—violet). The axes notes include the percentage of total variance explained by the respective principal coordinate.

In general, microbiome composition was associated neither with creatinine nor with the urea levels (dbRDA, Bray-Curtis diversity metric, *p* > 0.05) (Figure 2). There were no significant correlations between the uremic markers (creatinine and urea) and the alpha-diversity of the bacterial community (Shannon diversity index, Spearman correlation, *p* > 0.05).

**Figure 3:**
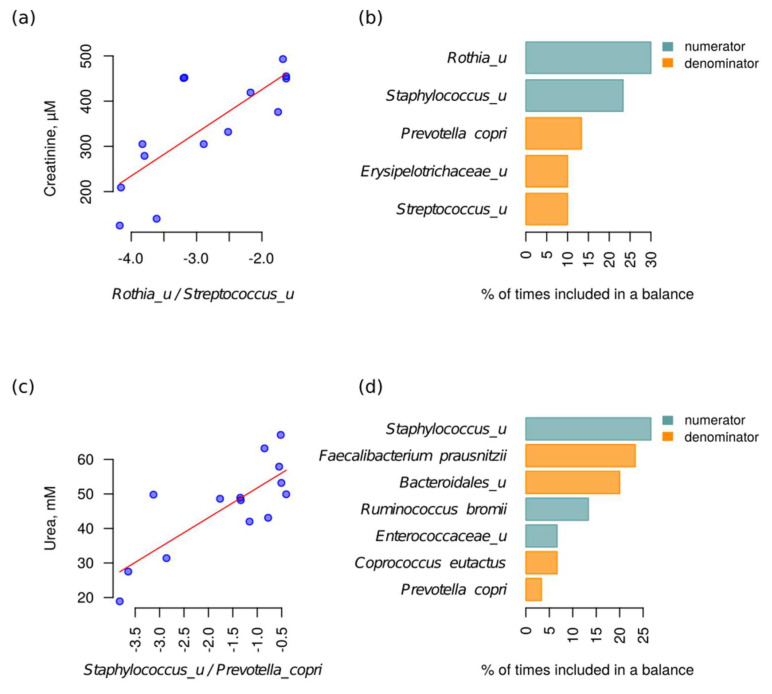
Bacterial balances associated with blood creatinine and urea values. (**a**,**c**) Linear regression between bacterial balances and metabolite values (a—creatinine, c—urea). The balances that were the best predictors in the analysis of the entire dataset are shown in the figure. (**b**,**d**) The occurrence of taxa among balance numerators or denominators (b—creatinine, d—urea). The members of 3 balances that were most frequent during the cross-validation procedure are shown. Designation “_u” denotes the unclassified species from the corresponding taxa.

The associations between bacterial abundance and metabolite levels were examined using a compositionality-aware approach [28]. According to this approach, the log-ratios of bacterial abundance (balances) were used as predictors rather than the relative abundance values themselves. The log-ratio between the presence of the *Rothia* and *Streptococcus* genera was found to be the best predictor of creatinine value (*p* = 0.0014, adjusted R^2^ = 0.55). A similar association was observed at the species level—the best predictor was the ratio between the unclassified species from the two above-mentioned genera. Moreover, the *Rothia* abundance was selected as a balance numerator in >25% iterations of the cross-validation procedure. Taken together, these two observations indicate a possible positive association between *Rothia* abundance and creatinine level (Figure 3). In addition to the discovered balance between *Rothia* and *Streptococcus*, which was the best predictor in the analysis including the full dataset, a few taxa were included in the top 3 most frequent balances during cross-validation—unclassified species from the Staphylococcus genus as the numerator and unclassified species from the *Erysipelotrichaceae* family and the *Streptococcus* genus as the denominator (Figure 3).

The best balance to predict blood urea values was the balance between unclassified species from the *Staphylococcus* genus and *Prevotella copri* (*p* = 0.0006, adjusted R^2^ = 0.60). Similarly, on the level of genera, the best predictor was the log-ratio between *Staphylococcus* and *Prevotella*. The *Staphylococcus* abundance was selected as the numerator of the balance in >25% of cross-validation iterations (Figure 3). Thus, this implied a possible positive association between *Staphylococcus* abundance and urea concentration. The list of taxa included in the top 3 balances during cross-validation also included *Ruminococcus bromii* and unclassified *Enterococcaceae* as the numerator and *Faecalibacterium prausnitzii*, *Coprococcus eutactus* and unclassified species from the *Bacteroides* genus as the denominator (Figure 3).

### 2.3. Metabolome and Microbiome during AKI

In addition to the “microbiome–AKI severity” axis (with creatinine and urea as the markers of the latter), we evaluated correlations between blood metabolites (excluding uremia-associated creatinine and urea) and the gut microbial community structure. For dimensionality reduction, the metabolites were initially clustered into highly correlated groups (n = 15, Spearman correlation coefficient > 0.7); see Supplementary Table S2. For each cluster, the associations with the microbiota composition were analyzed using the same method of balances as for the AKI severity. Six metabolite clusters significantly associated with the microbiome were singleton (i.e., including a single metabolite) (Table 2).

## 3. Discussion

**Figure 4:**
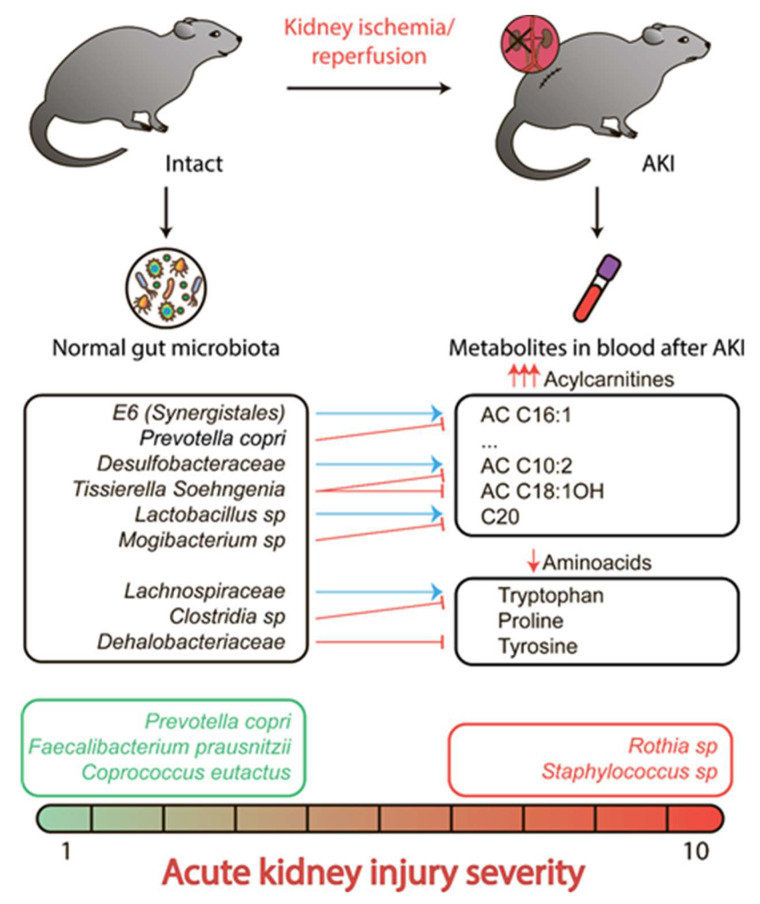
Elevated levels of acylcarnitines and drop of three amino acids concentrations in serum after renal ischemia/reperfusion and its associations with some bacterial clades (blue arrows indicate positive correlation, and red arrows indicate negative correlation). The analysis of bacterial balances revealed that *Prevotella copri, Faecalibacterium prausnitzii*, and *Coprococcus eutactus* prevalence was associated with low creatinine and urea levels, whereas *Rothia* and *Staphylococcus* positively correlated with severe acute kidney injury.

Levels of creatinine and urea in the blood are believed to be a “gold standard” for assessment of kidney function and detection of AKI in clinical practice [29,30]. The blood concentrations of these compounds (so-called uremic toxins) are directly linked to kidney function impairment [31]. In our study, we used the level of serum creatinine as a marker of AKI severity. After I/R, creatinine concentration increased in all animals, varying from 50 to 200–500 µM, indicating that I/R caused AKI. In our study, we were interested in interpreting the extensive intra-subject variability of post-I/R creatinine concentration. Although during renal I/R many parameters can affect the severity of AKI, there are some reasons to consider microbiome as one of the possible factors playing a role in this issue [32,33]. All our main findings are summarized in Figure 4.

There is growing evidence that microbiota is directly or indirectly involved in the regulation of a large number of organism functions [34]. In particular, the normal microbiome protects against pathogenic microorganisms, participates in the synthesis of essential substances, and modulates endocrine, neural, and immune systems [35,36,37,38]. An individual microbiome harbors microbial species that range in their effects of host health—from beneficial and commensal to opportunistic and possibly pathogenic. For example, some bacteria (including Gram-negative LPS-containing) can increase intestinal permeability, thereby leading to penetration of harmful antigens to the bloodstream [39,40]. To date, there were only a few attempts to analyze bacterial taxa that could be considered potentially nephroprotective or, conversely, causing aggravation of kidney damage [41,42].

In this study, using a compositionality-aware approach to microbiome data analysis, we discovered a few associations between microbiome and urea or creatinine levels after kidney I/R. From our results, it could be concluded that *Rothia* and *Staphylococcus* were likely associated with the severity of kidney damage, i.e., they not only demonstrated a noticeable positive correlation (as a balance numerator) with the rise of creatinine or urea concentration and, thus, AKI severity, but they could also be selected as a numerator of the balances in >25% of cross-validation procedure iterations. On the other hand, the denominators of these balances were not very stable—none of the taxa were selected as a denominator in >25% of iterations, for creatinine or for urea. However, the majority of denominator candidates were commensal rat intestinal bacteria—*Prevotella copri*, *Faecalibacterium prausnitzii*, *Coprococcus eutactus*, unclassified *Bacteroides*, and *Streptococcus*. Presumably, while we could identify the gut microbial taxa-determinants of kidney damage, the concept of "nephroprotectors" was less specific and encompassed the rat reference gut microbiome that could be driven by a completely different set of commensal taxa. 

From our analysis of microbiome and metabolome, we could propose some ways through which bacteria affect kidney damage. For instance, it was shown that bacteria play a “nephroprotective” role (*Prevotella copri* [43], *Faecalibacterium prausnitzii* [44,45], and *Coprococcus eutactus* [46]), being reversely associated with the severity of AKI, and are known to produce short-chain fatty acids (SCFAs), mainly represented by acetate, propionate, and butyrate [47]. Most of these molecules (especially butyrate) are used by the intestine epithelium as an energy source, however, a fraction of these goes into the bloodstream, and then transfer to the organs, inhibiting the histone deacetylase activity [48]. SCFAs regulate cell proliferation and differentiation, hormone secretion, and inflammatory response [24]. These molecules potentially mediate the nephroprotective effects of the mentioned bacteria since the treatment of I/R animals with such SCFAs has a positive effect through a reduction of the severity of AKI [13,23]. SCFAs reduce the infiltration of leukocytes into a damaged tissue and also affect chemotaxis and cytokine production [41]. Thus, elimination of these bacteria can result in a decrease of SCFAs production and reduce the positive effects on kidney tolerance.

The relationships between disease onset and progression with gut microflora have recently been reported, with ever-increasing clarity and importance. In humans, decreased levels of *Prevotella copri* and *Faecalibacterium prausnitzii* populations were observed in severe forms of chronic kidney disease (CKD), which also negatively correlate with the presence of important diagnostic markers, such as C-reactive protein and cystatin C levels [49,50,51]. Similarly, *Prevotella copri* and *Faecalibacterium prausnitzii* were significantly depleted in humans with diabetic nephropathy [43,52]. Conversely, the symptoms of diabetes and inflammation were also shown to be ameliorated upon the administration of *Faecalibacterium prausnitzii* to such patients [53], suggesting an important diagnostic and therapeutic relevance of these microorganisms. Additionally, reduced *Faecalibacterium prausnitzii* levels in the gut have also been observed during the prevalence of ulcerative colitis and Crohn’s disease [54,55,56]. Furthermore, in patients with Parkinson’s disease, the gut levels of *Prevotella copri*, *Faecalibacterium prausnitzii,* and *Coprococcus eutactus* were also observed to be relatively decreased [57]. Collectively, such reports demonstrate the significance of gut microflora and the relative associated contribution of certain species to a disease progression. However, how such findings resonate with the results observed in our rat model of AKI remains unknown, as the species of *Prevotella copri* and *Faecalibacterium prausnitzii* were observed to be quantitatively reduced. Although the human gut microbiome might differ significantly from that in rat, due to differences in the relative abundance of many bacterial clades, a considerable fraction (not limited to major phyla) and many common genera are, however, extensively shared [58]. Consequently, this allows us a greater scope to extrapolate the findings from our working rat model towards a human context, with a view to dissect the potential relationship between the human microbiome and AKI/CKD progression, with proper accuracy and effect.

As the above-mentioned studies revealed that after onset of a pathological condition, the levels of bacteria with “protective” potential were decreased, we attempted to establish what bacterial composition prior to the damage might serve as a predictor of severity of pathology. However, it is likely that the association between pathological conditions and gut microbiome presents a possibility of a "vicious circle". Bidirectional relationships between the host organism and its intestinal microbiota have been explored [8]. As the microbiota could affect the disease, the disease could change the composition of the gut microbiota, indicating the complexity and vulnerability of such interactions [10].

In addition, in the numerators of the balances for urea and creatinine, bacteria that are normally associated with the microbiota of parts of the organism other than the gut prevail. For example, *Rothia* and *Staphylococcus* are facultative aerobes and typical representatives of the nasopharynx or skin microbiota, in humans [59], as well as in rats [60]. Conversely, typical representatives of the intestinal microbiota *Faecalibacterium prausnitzii*, *Prevotella copri* (obligate anaerobes), *Erysipelotrichaceae*, and Bacteroidales prevailed in the denominators of the observed balances [61]. In this regard, we could assume that the levels of AKI severity correlate with the intensity of translocation of the bacteria from the oral cavity to the intestine. The phenomena of translocation of oral microbes were observed, for example, with a decrease in the acidity of the stomach as a result of taking medications [62] or alcohol-induced liver cirrhosis [63]. The only exception could be considered for *Streptococcus*, which is negatively associated with AKI severity and, along with being the part of the normal rat intestinal microbiota [64], it can also dwell in the oral cavity of people and animals [65,66].

Analyzing a metabolomic profile of blood serum after AKI, we found a significant increase in levels of many acylcarnitines. The conventional explanation is an impaired secretion of these metabolites [67]. Not surprisingly, such an increase was highly correlated with AKI severity—the more that the kidney function was impaired, the more these compounds accumulated in the blood. The ratio of acylated carnitines to free carnitine is an important diagnostic parameter for a number of diseases [68].

Nonetheless, the increase of acylcarnitines levels could have different effects on the organism. Elevated concentrations of long-chain acylcarnitines and their CoA esters are suggested to be hazardous [69]. In ischemic tissue, long-chain acylcarnitines accumulate at high concentrations and are thought to inhibit oxidative phosphorylation, induce mitochondrial membrane hyperpolarization, and increase production of reactive oxygen species [70]. Long-chain acylcarnitines inhibit citrate lyase and increase the activity of citrate synthase, thereby, resulting in an elevation in cytosolic citrate concentrations, enhanced acetyl-CoA carboxylase and carnitine palmitoyltransferase activity, and increased malonyl-CoA concentrations, thus, inhibiting mitochondrial fatty acid oxidation [69]. Moreover, long-chain acylcarnitines have been shown to act as detergents and, therefore, disrupt lipid membranes [71]. After AKI, elevated concentrations of acylcarnitines in serum are accompanied by elevated levels of acylcarnitine esters [72], which are shown to increase intracellular calcium [73].

While long-chain acylcarnitines have been linked to poor clinical status, many studies have observed that short-chain acylcarnitines are associated with positive effects [74,75]. Free carnitine is believed to have a protective effect by removing long-chain acyl CoAs from cell membranes, thereby, stabilizing them [68]. L-carnitine, acetyl-carnitine, propionyl-L-carnitine, and other short-chain acylcarnitines are believed to be beneficial in the treatment of various disorders, including different kidney diseases, via increased carnitine content in the mitochondria and stimulation of the Krebs cycle [76,77,78,79,80]. We detected elevated concentrations of free carnitine and short-chain acylcarnitines in rats’ serum, 48 hours after renal I/R, which potentially could be a protective response of tissue to the damage. It should be noted that acyl-carnitines are closely related to energy metabolism and mitochondria function [81]. Creatinine is a product of degradation of creatine phosphate [82], one of the main energy substrates in mitochondria [83]. Therefore, the correlation between the levels of these metabolites might reflect the involvement of mitochondria in metabolic changes after AKI, which was previously indicated for a number of uremic toxins [84].

A revealed drop in the concentrations of 3 amino acids—tyrosine, tryptophan, and proline—seems to be an interesting finding, indicating certain functional alteration. A tyrosine blood drop has been earlier observed during CKD and was explained as an impaired synthesis of tyrosine from phenylalanine, by the kidney [85]. We also observed such a drop in our AKI model, which was accompanied by an increase in blood phenylalanine concentration, confirming the disruption of tyrosine synthesis. Indeed, we observed a strong negative correlation between AKI severity and tyrosine concentration (–0.5) and a similar strong positive correlation between AKI and phenylalanine/tyrosine ratio (+0.5). We have also found an association of unclassified *Dehalobacteriaceae*, negatively correlating with tyrosine concentration. The observed tryptophan concentration decay was also described earlier in human CKD patients [86]. There was a strong negative correlation (r = –0.6) between tryptophan concentration and AKI severity. Unclassified *Lachnospiraceae* were found to be positively correlated with tryptophan concentration, whereas, unclassified Clostridia were correlated negatively. For the first time, an association between impaired kidney function and a decrease of serum proline were detected. Moreover, among all metabolites for which their concentration decreased during AKI, proline demonstrated the highest shift. One of the explanations for this could be a decreased reabsorption of proline in kidney, coupled with an elevation concentration of 5-Oxo proline [87].

We also revealed certain other associations of microbiome composition with blood metabolites (Table 1). Interestingly, several associations included sulfate-reducing bacteria (SRB). In our study, the relative abundance of SRB (*Desulfobacteraceae*) was associated positively with metabolites related to AKI severity Decadienoylcarnitine. In the gut, members of *Desulfobacteraceae* utilize H_2_ to produce hydrogen sulfide [88]. A number of studies have shown that endogenous hydrogen sulfide is involved in many important biological processes, in particular, in the regulation of blood pressure, the functioning of the kidneys, the heart, and the brain [88,89,90]. It has also been shown that hydrogen sulfide produced by the members of the intestinal microbiota can cause an effect on the circulatory system in the same way as endogenous hydrogen sulfide [88]. The role of hydrogen sulfide in chronic and acute kidney disease is considered to be context-dependent [89,90]. The protective role of the metabolite was shown in models of ischemia-reperfusion and obstructive kidney injury [90], as well as in kidney impairments caused by diabetes and renovascular hypertension [91,92]. On the other hand, controversial data were obtained on the role of H_2_S, when using nephrotoxic cisplatin [89]. Both the protective anti-inflammatory and aggravating proinflammatory role of this metabolite has also been demonstrated in many animal models of pathologies, although it might be a question of concentration of this agent [93].

## 4. Materials and Methods 

### 4.1. Animals

Experiments were performed on male outbred Wistar rats (3–4-month old, 300–400 g weight). The analysis included samples from 14 rats subjected to ischemia/reperfusion (I/R) and 6 intact animals. Animal experiments were evaluated and approved by the animal ethics committee of the Belozersky Institute—Protocol 3/19 from 18 March 2019. All procedures were in accordance with the Federation of Laboratory Animal Science Associations (FELASA) guidelines.

### 4.2. Kidney I/R Protocol

For the I/R, rats were anesthetized with chloral hydrate (300 mg/kg, i.p.) and subjected to 40-min warm ischemia of the left kidney, as previously described [27]. In brief, the renal vascular bundle was occluded with a non-traumatic microvascular clip, for 40 min. Circulation was restored by removing the clip; the lack of blood flow during ischemia and its restoration during reperfusion were assessed visually. The nephrectomy of the right kidney was performed, simultaneously, with ischemia of the left one. During surgery, the body temperature of the rats was maintained at 37 ± 0.5 °C. Blood samples were taken 48 h after I/R from the carotid artery to determine SCr, BUN, amino acids, and acylcarnitines concentrations. Levels of SCr and BUN were analyzed using the AU480 Chemistry System (Beckman Coulter, Brea, CA, USA).

### 4.3. Tandem Mass Spectrometry

The analysis of amino acids and acylcarnitines in the rat serum was performed 48 h after I/R by FIA–MS/MS analysis, using a NeoGram Amino Acids and Acylcarnitines Tandem Mass Spectrometry Kit (Perkin Elmer Life and Analytical Sciences, Waltham, MA, USA) and a Sciex QTrap 3200 (Sciex, Framingham, MA, USA) quadrupole tandem mass spectrometer, operating with the positive electrospray ionization technique, coupled with Shimadzu 20LC system (Shimadzu, Japan).

Amino acids and acylcarnitines were extracted from 5 µl plasma, with a methanol/water (75:25) solution containing stable isotope-labeled internal standards. The samples were diluted in butanolic HCl, dried at 60 °C, and reconstituted with acetonitrile/water (80:20) solution containing acetic acid. A 20 µl aliquot of the sample was directly injected into the MS/MS system.

The analyte concentrations were measured by comparing the instrument responses for each amino acid and acylcarnitine with the responses for the corresponding stable isotope-labeled internal standards. The concentrations of amino acids and acylcarnitines were calculated automatically using the ChemoView software version 2.0.2 (Sciex, Framingham, MA, USA).

### 4.4. DNA Extraction and Sequencing

DNA extraction from rat fecal samples and library preparation was performed, as described in [94]. The V4 region of the 16S rRNA gene was amplified using the following modification of 515F–806R primers: GTGBCAGCMGCCGCGGTAA and GACTACNVGGGTMTCTAATCC. The libraries were sequenced on an Illumina MiSeq. The reads were deposited in the European Nucleotide Archive (ENA), under accession number PRJEBxxxx.

### 4.5. Bioinformatic Analysis

Data analysis was performed in the Knomics-Biota system [95]. Briefly, the reads were denoised using the DADA2 algorithm [96] with the variable trimming length (from 251 to 253 bp). Then, the taxonomic classification of the denoised reads was performed with the QIIME2 naive-bayes classifier [97] and GreenGenes database [98]. For alpha- and beta-diversity analysis, the classified reads were randomly rarefied to the same number (3000 reads per sample), for each sample. Estimation of alpha-diversity for each sample was performed using the Shannon diversity metric. Beta-diversity (pairwise dissimilarity between the gut community structures) was estimated using a Bray-Curtis dissimilarity metric. Read counts of microbial species, genera, and families were calculated as the sum of reads assigned to the ASVs (amplicon sequencing variants) belonging to the respective taxon.

Analysis of the association between general microbiome composition and metabolite levels was performed using dbRDA (adonis R function) [99]. Analysis of correlation between alpha-diversity and metabolite levels was performed using Spearman correlation. For all metabolites except urea and creatinine, the multiple comparison adjustment was performed using the Benjamini–Hochberg method for alpha- and beta-diversity analysis.

The analysis of associations between bacteria abundance and metabolite levels was performed by applying the compositionality-aware approach—selbal [28]. The approach allows one to find associations between a factor of interest and bacterial balances—normalized log-ratios of the bacteria abundances geometric means for two groups of bacteria (numerator and denominator). The algorithm was applied to non-rarified abundance tables. The algorithm predicted the optimal number of bacteria in groups, found the best balance to predict the variable of interest, and applied a cross-validation procedure to assess the stability of numerator and denominator members. We considered the association to be significant if the *p*-value from the linear regression analysis between the balance and the factor was less than 0.05 after the multiple comparison correction (Benjamini–Hochberg) and, at the same time, the association was relatively stable (the bacteria was selected as a part of the balance in more than 25% of cross-validation iterations). For the urea and creatinine levels, the adjustment for multiple comparison was not performed, as these were the variables of dedicated interest.

## 5. Conclusions

We identified the main members of rat intestinal microbiota whose balances were correlated in rats, with the severity of renal dysfunction measured by serum creatinine and urea. Correlations between microbiome composition and bacterial metabolites were observed. The results highlight the specific taxa that could confer «nephroprotective» or «nephropathogenic» activity in the gut. Further experiments involving the transfer of fecal microbiota or these specific taxa would allow validation of these findings.

## Figures and Tables

**Table 1:** Statistically significant changes in metabolites concentration after acute kidney injury (AKI) and their Pearson correlation coefficient with the creatinine concentration. The grey gradient indicates the value of AKI/control metabolite levels ratio, and the blue color gradient indicates the strengths of the metabolite/creatinine levels correlation.

Metabolite	AKI vs. Control				Correlation with SCr
	AKI/Control	FDR Adjusted *p*-Value	Mean in AKI, µM	Mean in Control, µM	
Malonylcarnitine (AC C3DC)	6.71	0.00073	0.244	0.036	0.89
Glutarylcarnitine (AC C5DC)	4.55	0.00301	0.253	0.056	0.76
Decadienoylcarnitine (AC C10:2)	4.30	0.00073	0.016	0.004	0.72
3-hydroxybutyrylcarnitine (AC C4OH)	3.85	0.00432	0.061	0.016	0.63
Linoleylcarnitine (AC C18:2)	3.76	0.00128	0.082	0.022	0.63
Methylmalonylcarnitine (AC C4DC)	3.51	0.00301	0.154	0.044	0.85
Hexanoylcarnitine (AC C6)	3.41	0.00073	0.046	0.014	0.75
Acetylcarnitine (AC C2)	3.15	0.00167	26.116	8.296	0.77
Octanoylcarnitine (AC C8)	3.13	0.00081	0.021	0.007	0.86
Oleoylcarnitine (AC C18:1)	3.08	0.00002	0.123	0.040	0.79
3-hydroxystearylcarnitine (AC C18OH)	2.75	0.00891	0.023	0.008	0.73
3-hydroxyoleylcarnitine (AC C18:1OH)	2.70	0.00891	0.020	0.007	0.51
3-hydroxypalmitoylcarnitine (AC C16OH)	2.44	0.01884	0.021	0.009	0.74
Hydroxyhexanoylcarnitine (AC C6OH)	2.41	0.00573	0.026	0.011	0.71
Arachidylcarnitine (C20)	2.39	0.00228	0.035	0.015	0.32
Palmitoylcarnitine (AC C16)	2.32	0.00073	0.236	0.102	0.70
Stearoylcarnitine (AC C18)	2.30	0.00073	0.132	0.058	0.66
3-hydroxypalmitoleylcarnitine (AC C16:1OH)	2.25	0.01022	0.023	0.010	0.54
Tetradecadienoylcarnitine (AC C14:2)	2.23	0.00108	0.045	0.020	0.72
Tetradecenoylcarnitine (AC C14:1)	2.16	0.00432	0.101	0.047	0.69
Palmitoleylcarnitine (AC C16:1)	2.14	0.00482	0.052	0.024	0.66
3-hydroxyisovalerylcarnitine (AC C5OH)	2.12	0.00827	0.092	0.043	0.73
Butyrylcarnitine (AC C4)	2.10	0.01656	0.455	0.216	0.57
Octenoylcarnitine (AC C8:1)	2.09	0.00223	0.012	0.006	0.81
Adipylcarnitine (AC C6DC)	2.03	0.00991	0.096	0.047	0.77
Myristylcarnitine (AC C14)	2.00	0.00281	0.095	0.047	0.74
Dodecanoylcarnitine (AC C12)	2.00	0.00159	0.124	0.062	0.61
Decanoylcarnitine (AC C10)	1.96	0.00788	0.033	0.017	0.82
Decenoylcarnitine (AC C10:1)	1.81	0.01221	0.027	0.015	0.65
3-hydroxymyristylcarnitine (AC C14OH)	1.76	0.03478	0.012	0.007	0.67
Free carnitine (AC C0)	1.72	0.01656	51.941	30.283	0.64
Tyrosine (AA Tyr)	0.73	0.00573	57.410	78.415	-0.54
Tryptophan (AA Trp)	0.66	0.01200	11.244	16.991	-0.61
Proline (AA Pro)	0.60	0.00223	114.723	192.781	-0.70

**Table 2:** Significant associations between blood metabolites and bacterial abundance. NUM—numerator, DEN—denominator.

Metabolite	Taxon	*p*-Value, adj.	R^2^, adj.	Position in a Balance	% of Times Included in a Balance
Hexadecenoylcarnitine (AC C16:1)	unclassified *E6* (Synergistales)	0.00028	0.74793	NUM	50
	*Prevotella copri*			DEN	56
Tryptophan	unclassified *Lachnospiraceae*	0.00028	0.72956	NUM	37
	unclassified Clostridia			DEN	43
Decadienoylcarnitine (AC C10:2)	unclassified *Desulfobacteraceae*	0.00028	0.73393	NUM	33
	unclassified *Tissierella*/*Soehngenia*			DEN	33
Arachidylcarnitine (C20)	unclassified *Lactobacillus*	0.00045	0.69365	NUM	33
	unclassified *Mogibacterium*			DEN	30
Tyrosine	unclassified *Dehalobacteriaceae*	0.00135	0.6112	DEN	33
Hydroxyoleoylcarnitine (AC C18:1OH)	unclassified *Tissierella*/*Soehngenia*	0.00617	0.46336	DEN	26

## Supplementary Materials

The following are available online at https://www.mdpi.com/2218-1989/10/4/142/s1, Supplementary Figure S1: Structures and pathological conditions that associate with acylcarnitines. Supplementary Table S1: The whole dataset of metabolites concentration in rats’ serum. Supplementary Table S2: Whole dataset of associations between blood metabolites and bacterial abundance.
