# Supplementary material for: Microbiome-Metabolome Signature of Acute Kidney Injury
Source: Metabolites. 2020 Apr 4;10(4):142. doi: 10.3390/metabo10040142 (PMC7241241; doi:10.3390/metabo10040142)
Supplement: Supplementary file 1 [file metabolites-10-00142-s001.zip › Supplementary figure 1.docx]

**Supplementary figure 1.** Structures and pathological conditions that associate with 5 acylcarnitines, which both demonstrate the greatest elevation in acute kidney injury and have the strongest correlation with creatinine concentration.
